# Comparative Effects of Fatiguing Exercise on Anticipatory and Compensatory Postural Adjustments between Trained and Untrained Individuals

**DOI:** 10.3390/life14080943

**Published:** 2024-07-28

**Authors:** Hui Lyu, Xueying Cao, Jian Wang

**Affiliations:** 1 Ningbo Innovation Center, Zhejiang University, Ningbo 315100, China; lvhui@zju.edu.cn; 2 Faculty of Sports Science, Ningbo University, Ningbo 315211, China; 3 Department of Sports Science, Zhejiang University, Hangzhou 310058, China

**Keywords:** anticipatory postural adjustments, compensatory postural adjustments, postural stability, comprehensive training, fatigue, electromyography

## Abstract

This study evaluates the effects of general fatiguing exercises on anticipatory postural adjustments (APAs), compensatory postural adjustments (CPAs), and standing stability between 18 individuals with comprehensive training experience (TR) and 18 untrained individuals (UT). Assessments were conducted before and after a 20-min fatiguing exercise using surface electromyography and a force platform during self-initiated perturbation and postural stability tests. Key findings include that, irrespective of fatigue, the APAs onsets in the TrA/IO (*p* = 0.004), LMF (PRE *p* = 0.003, POST *p* < 0.001), and ST (PRE *p* = 0.001, POST *p* = 0.006) muscles activated earlier in the TR group than in the UT group. Additionally, the APA co-contraction indices of the TrA/IO-LMF (PRE *p* = 0.011, POST *p* = 0.029), TrA/IO-ST (*p* = 0.014), and LMF-ST (PRE *p* = 0.002, POST *p* = 0.005) muscle pairs were higher in the TR group. After fatigue, the UT group significantly increased CPA co-contraction indices for the TrA/IO-LMF (*p* = 0.035) and LMF-ST (*p* = 0.005) muscle pairs. This research highlights the importance of comprehensive training in facilitating feedforward control strategies, particularly for individuals facing challenging postural conditions, such as fatigue or disturbances.

## 1. Introduction

Efficient control of body posture is fundamental to skillful sports performance [1]. For example, in sports like soccer and judo, successful movement execution critically depends on the preparatory positioning of the trunk and lower legs, which is guided by anticipating the trajectory of the ball or an opponent’s movements [2]. Anticipatory postural adjustments (APAs) refer to the activation of postural muscles through a feedforward mechanism prior to voluntary movements or external disturbances [3]. However, due to discrepancies between predicted information and actual outcomes, compensatory postural adjustments (CPAs) are initiated by sensory feedback after disturbances to work in coordination with APAs to maintain postural stability.

Exercise-induced muscle fatigue is an overlooked cause of falls and injuries [4]. It not only decreases receptor sensitivity and central contribution rates but also impairs muscle contraction functionality [5,6]. Additionally, it slows the transmission of sensory information and motor commands [7,8] and reduces the efficiency of central integration and information processing [9], thereby impacting postural control. As a crucial component of postural control, the influence of muscle fatigue on APAs and CPAs is still debated. Early theories, such as the Similar Impulse Hypothesis [10] and the Critical Force Hypothesis [11], from a view of local muscle compensation strategies, suggested that postural muscles advance the APAs onset after fatiguing exercise to maintain activation levels similar to pre-fatigue conditions, thereby ensuring body balance. Later studies have favored the General/Common Response Hypothesis, taking a holistic central compensation view. One of its representative studies reported delayed onset and reduced amplitude of APAs in proximal postural muscles after fatiguing exercise while increasing the co-activation amplitude of APAs in distal posture muscles to compensate for fatigue effects [12]. Our research has demonstrated that APA onsets in trunk postural muscles are advanced following unilateral and bilateral lower extremity fatiguing exercises [13]; the co-activation amplitude of APAs and CPAs in trunk muscle pairs increases after local and general fatiguing exercises [14]. However, research into the patterns and mechanisms by which fatiguing exercises affect APAs and CPAs remains to be elucidated.

Given the importance of APAs and CPAs in sports and rehabilitation, how training exercises can enhance their responsiveness under fatigue or illness has attracted growing interest. Research suggests that highly trained individuals can effectively prioritize detected sensory information and adapt the sensory frame of reference to regulate postural balance under challenging or disturbed conditions [15]. Concurrently, APAs and CPAs, which respectively generate reticulospinal and vestibulospinal reflexes at the subcortical level, are proposed to be refined, and the contribution of cerebellar structures involved in postural regulation is augmented with training [16]. However, the impact of training on postural performance is sport-specific, and even athletes engaged in the same sport can exhibit different postural control advantages due to their varying roles or positions within the sport. For example, judokas demonstrated better trunk responses against lateral and posterior loading, while kayakers showed better trunk balance control while sitting on an unstable seat [17]. Defensive soccer players develop their CPAs to counteract opponents’ attacking moves, while offensive players enhance their APAs due to their proactive and creative roles [16]. Similarly, short-term specialized training exercises have shown beneficial effects for patients or individuals without a sports background. A brief 20-min ball-catching exercise session with 130 repetitions advanced APAs onsets in six lower limb postural muscles during a pendulum test, mirroring the movement of ball-catching [18]. Another study reported significant advancements in APA onsets in the thoracic and lumbar erector spinae following an 8-week boxing training program [19]. The study employed rapid arm lift tests to induce APAs, closely mimicking boxing movements. The above findings indicate that specific technical actions in sports practice, when repeated regularly and frequently, develop targeted postural skills associated with specific motor skills [16]. Research by Barbado D, Barbado L C, Elvira J L, et al. [17] suggests that detecting postural adaptations from training requires specific assessment tests tailored to relevant movements. This stems from the hypothesis that exclusive repetition of specific postures and movements leads to distinct postural adaptations [20]. Similarly, it is implied that postural adaptations resulting from specific and long-term training, which require particular support positions and body segment placements, barely transfer to untrained or inexperienced postural and motor tasks [21].

What if the training exercises focus on fundamental capabilities such as speed, endurance, and strength, but are not specific to the task tested? Can training in these basic abilities facilitate the activation of APAs and CPAs under conditions of fatigue? Therefore, the primary objective of this study is to explore the performance of APAs and CPAs before and after fatiguing exercise in individuals with comprehensive professional training experience compared to those without any training experience. Specifically, this study investigates whether fundamental and comprehensive training experience/ trained status can facilitate the responsiveness of APAs and CPAs under fatigue conditions, thereby providing theoretical evidence on the application of basic training regimens in enhancing preparatory and compensatory postural strategies in a state of fatigue. We hypothesize that individuals with comprehensively trained status will demonstrate advanced activation patterns in APAs and CPAs compared to those who are untrained, regardless of fatigue conditions.

## 2. Methods

### 2.1. Participants

This study recruited 36 male university students from Zhejiang University, including 18 in the trained group (TR) and 18 in the untrained group (UT). The inclusion criteria for the TR group, all from the Department of Sports Science, required participants to hold Chinese national second-level athlete certifications and have at least two years of comprehensive athletic training experience. This training followed a standardized national curriculum, including the 100-m sprint, shot put, standing long jump, and 800-m run, corresponding respectively to speed, strength, explosive power, and endurance. The UT group consists of students from non-sports-related disciplines, recruited after confirming that they have no professional athletic training experience or regular fitness or exercise habits. Prior to participation, each participant was confirmed to be free from any cardiovascular or neurological disorders and had not experienced any musculoskeletal injuries or surgeries in the past six months. Subjects were asked not to consume caffeine, alcohol, or tea, to keep their diets consistent, to avoid intense exercise, and to ensure enough sleep for at least 24 h before the test. The experiments were conducted between 13:00 and 17:00, with each participant tested once; all tests were completed within two weeks. This experiment was approved by the Ethics Review Board of Zhejiang University. All the subjects provided informed consent prior to their participation.

### 2.2. Procedure

Both the TR and the UT groups followed the identical experimental protocol. After familiarizing themselves with the procedure and donning the necessary equipment, participants first conducted self-initiated perturbation tests (fast arm lifts) and postural stability tests. This was followed by a 20-min rowing fatiguing exercise, after which they immediately repeated both the perturbation and postural stability tests. Heart rate (HR) and Borg scale ratings for perceived exertion (RPE, 6–20) were recorded in pre- and post-fatigue sessions. The rowing ergometer was positioned within 3 m of the assessment area to minimize transition time. The entire session lasted approximately 30 min.

### 2.3. Self-Initiated Perturbation Test

Participants stood barefoot with feet together, grasping a 2 kg, 43 cm long iron bar at their sides with arms fully extended as the starting position. They focused on a circular wireless-controlled light positioned 1.5 m ahead on a wall. Upon seeing the light being turned on, participants were required to quickly flex their shoulders until their arms were parallel to the ground. When the light is turned off, they should gradually return their arms to the starting position and relax [13]. This visual signal was controlled by a fixed operator, with random intervals between signals. To ensure consistency in arm lifting speed and shoulder movement range, participants were allowed up to ten practice attempts before the official tests, after which each participant underwent three formal tests.

During the tests, surface electromyography (Trigno Wireless System, Delsys, Natick, MA, USA) recorded muscle activity from the right transversus abdominis/internal oblique (TrA/IO), lumbar multifidus (LMF), rectus femoris (RF), semitendinosus (ST), and anterior deltoid (AD) at a sampling rate of 2000 Hz. Electrode placement followed the SENIAM guidelines [22]. Before attaching the electrodes, the skin area was prepared by shaving, abrasion, and alcohol cleaning. These bipolar Ag/AgCl surface electrodes had an inter-electrode distance of 1 cm and dimensions of 10 × 1 mm^2^, with a common mode rejection ratio exceeding 80 dB.

### 2.4. Postural Stability Test

Participants stood upright, barefoot, feet together, with their hands relaxed at their sides, on a force platform (OR-6, AMTI, Watertown, MA, USA). During the test, participants were required to fix their gaze on a black cross marked on a wall 1.5 m in front of them and maintain a quiet and natural standing posture for 30 s. The force platform recorded the mediolateral (ML), anteroposterior (AP), and vertical components of the ground reaction force (FX, FY, and FZ), as well as the moments of the force around the frontal and sagittal axes (MX and MY), with a sampling rate of 1000 Hz. 

### 2.5. Fatiguing Exercise

The present study employed the same fatigue protocol as in previous research [14], which demonstrated significant effects on postural control, stability, and subjective fatigue perceptions. Notably, this fatiguing exercise was shown to exert a more pronounced impact on postural stability than the effects observed with isometric knee extension-based fatigue interventions. 

As not all participants were experienced with using a rowing ergometer, they watched a 5-min instructional video to learn the techniques of operating the rowing ergometer before starting the fatiguing exercise. Subsequently, under the guidance of an experienced coach, they performed a self-paced 1000-m rowing warm-up. After sufficient rest, the fatiguing exercise began, requiring participants to maintain a consistent rowing pace of 200 ± 5 m per minute for 20 min on a rowing ergometer (E, Concept 2, Morrisville, VT, USA). The damper setting on the ergometer was fixed at level 4. 

### 2.6. Data Analysis

**Figure 1:**
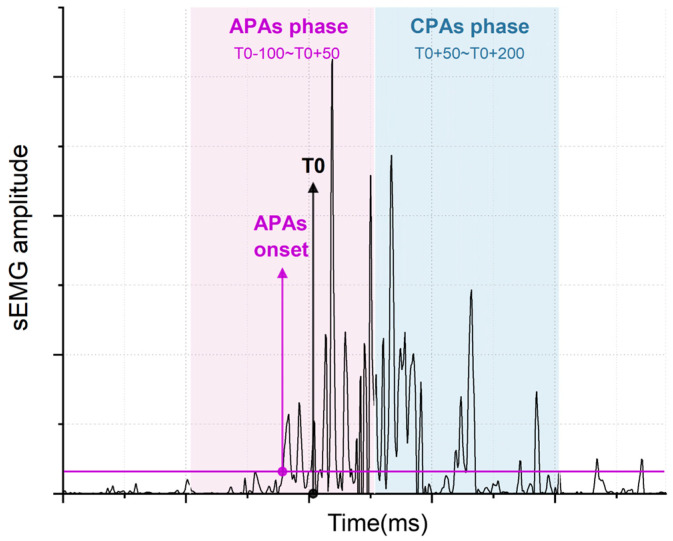
Schematic diagram illustrating the phases of Anticipatory Postural Adjustments (APAs) and Compensatory Postural Adjustments (CPAs).

Recorded data were processed offline using custom MATLAB programs (R2018a, MathWorks, Carlsbad, CA, USA). EMG data were first band-pass filtered at 20–450 Hz, then smoothed using the Teager–Kaiser energy (TKE) detection method [23], and fully rectified to determine muscle onset. Data before TKE analysis were further processed with a 100 Hz Butterworth low-pass filter (fourth-order, zero-lag) and full-wave rectification to compute integrated EMG (iEMG) within the APAs and CPAs windows. The onset of the anterior deltoid (AD) was marked as the beginning of the perturbation (labeled as T0). Figure 1 illustrates the method for dividing the APAs window (T0−100 to T0+50 ms) and CPAs window (T0+50 to T0+200 ms). The identification of postural muscle APAs onset required activation within the APAs window; data not activated within the APAs window were excluded. To ensure uniformity in the analysis, the iEMG values of APAs/CPAs were normalized across different muscles and participants as described in Equations (1) and (2). Additionally, the co-contraction indices for muscle pairs during both APAs and CPAs phases were calculated according to Equation (3). For detailed methods of data analysis, refer to Lyu H, Fan Y, Hua A et al. [13] and Lyu H, Fan Y, Hao Z et al. [14].

(1) $${Int}_{EMGi}=\int_{150}^{0}\mathrm{EMG}-\int_{-450}^{-600}\mathrm{EMG}\;$$

(2) $${iEMG}_{NORM}=\frac{{Int}_{EMGi}}{{iEMG}_{max}}$$

(3) $$co-contraction\;index=\int_{i=1}^{N}\frac{lower{EMG}_{i}}{higher{EMG}_{i}}\times \left(lower{EMG}_{i}+higher{EMG}_{i}\right)$$

### 2.7. Statistics

Sample size calculations were performed with G*Power v3.1 (Dusseldorf University, Germany), based on a moderate effect size (f = 0.2525) derived from a partial eta squared ($\eta_{p}^{2}$) of 0.06 [24]. The analysis indicated that 34 participants would suffice to achieve the desired power of 0.80 at an alpha level of 0.05. To strengthen the study’s robustness, 36 participants were ultimately recruited.

Statistical analyses were conducted using IBM SPSS Statistics 25. Independent sample t-tests were used to examine differences in age, weight, BMI, and training years between groups. Preliminary checks for normality and homogeneity of variances included Z scores for kurtosis and skewness and Levene’s test. With confirmed normal distribution and homogeneous variances, two-way repeated-measures ANOVAs were performed, focusing on the group (TR vs. UT) and fatigue condition (PRE: pre-fatigue vs. POST: post-fatigue). This analysis was applied to the RPE, HR, center of pressure (COP) variables, APA onset, APAs and CPAs iEMG, and co-contraction indices. Significant interactions were further analyzed using Bonferroni-corrected simple effects tests. Where criteria were not met, inter-group differences were assessed using the Mann–Whitney U test, and inter-condition differences were examined using the Wilcoxon signed-rank test. Statistical significance was set at *p* < 0.05. Data are presented as means ± standard deviations (SD). Effect sizes ($\eta_{p}^{2}$) and 95% confidence intervals were calculated for changes in dependent variables.

## 3. Results

All participants successfully completed the designated fatiguing exercise protocol. As demonstrated in Table 1, there were no significant differences between the TR and UT groups in terms of age (*p* = 0.41), height (*p* = 0.17), weight (*p* = 0.59), and BMI (*p* = 0.79). However, a significant difference was observed in the number of comprehensive training years (*p* < 0.001). 

### 3.1. RPE and HR

Table 2 displays the results of RPE and HR in both groups under PRE and POST conditions. RPE exhibited a significant main effect of fatigue (F_(1,34)_ = 285.521, *p* < 0.001, η^2^_p_ = 0.894) and a significant group-by-fatigue interaction effect (F_(1,34)_ = 31.196, *p* < 0.001, η^2^_p_ = 0.478). Simple effect analyses indicated a significantly higher RPE in the TR group compared to the UT group PRE (*p* < 0.001) and a significantly lower RPE in TR than in UT POST (*p* = 0.012). HR demonstrated a significant main effect of fatigue (F_(1,34)_ = 505.088, *p* < 0.001, η^2^_p_ = 0.937) and a significant effect of group (F_(1,34)_ = 14.183, *p* = 0.001, η^2^_p_ = 0.294). HR significantly increased in both groups following the fatiguing exercises. The TR group exhibited lower HR than the UT group, irrespective of fatigue conditions.

### 3.2. APA Onset

**Figure 2:**
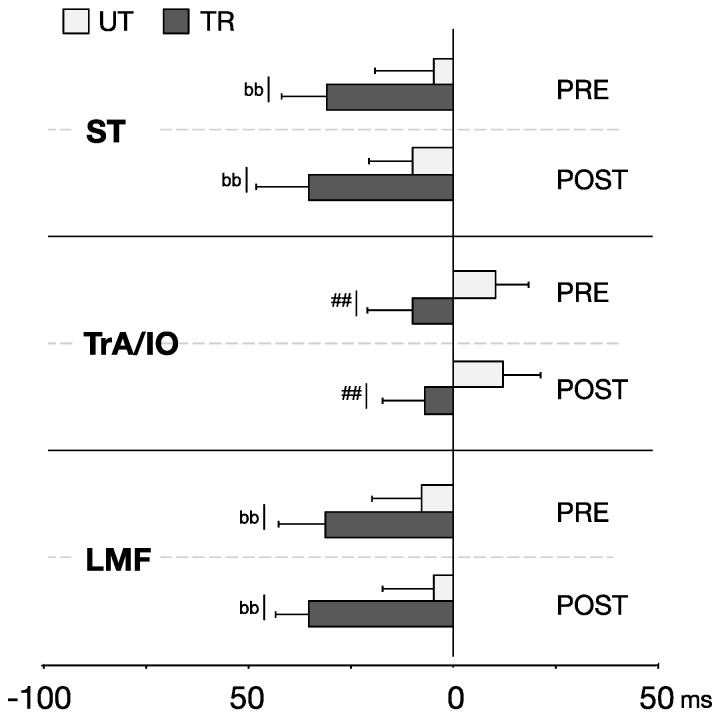
Onsets of APAs for individual muscles during self-initiated perturbation. Data are presented as mean ± SE. ‘##’ indicates a significant main group effect (*p* < 0.01). ‘bb’ denotes a significant difference between trained (TR) and untrained (UT) groups (*p* < 0.01).

Due to the low detection rate of APA onsets in the RF muscle, it was excluded from statistical evaluation [25]. APA onsets in both LMF and ST muscles did not follow a normal distribution. Results from Wilcoxon tests showed that the TR group demonstrated earlier APAs onsets in the LMF and ST muscles compared to the UT group, in both PRE (ST: *UT* −4.6 ± 29.5, *TR* −29.6 ± 22.9, *p* = 0.001; LMF: *UT* −7.5 ± 25.1, *TR* −30.2 ± 23.7, *p* = 0.003) and POST conditions (ST: *UT* −9.6 ± 22.2, *TR* −34.1 ± 26.6, *p* = 0.006; LMF: *UT* −4.5 ± 26.3, *TR* −34 ± 16.5, *p* < 0.001). Furthermore, a significant main group effect was observed for TrA/IO (F_(1,33)_ = 9.66, *p* = 0.004, $\eta_{p}^{2}$ = 0.226), with earlier onsets by the TR group compared to the UT group, as shown in Figure 2.

### 3.3. APAs and CPAs iEMG of Individual Muscles

**Figure 3:**
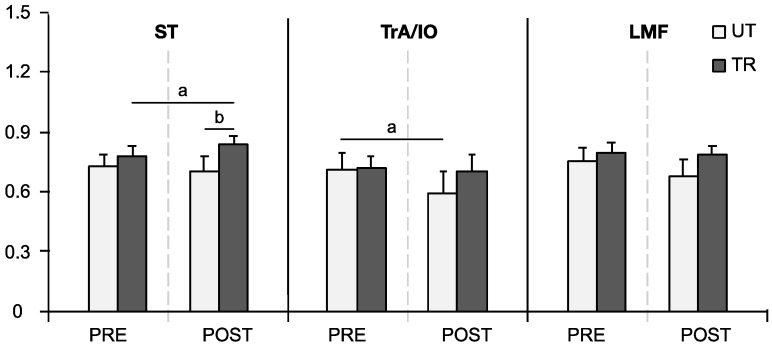
iEMG of APAs in individual muscles during self-initiated perturbation. Data are presented as mean ± SE. ‘a’ indicates a significant difference between pre-(PRE) and post-fatigue (POST) conditions (*p* < 0.05). ‘b’ denotes a significant difference between trained (TR) and untrained (UT) groups (*p* < 0.05).

Figure 3 illustrates that, based on Wilcoxon tests, APAs iEMG of the ST muscle in the TR group significantly increased after fatigue (*PRE* 0.779 ± 0.106, *POST* 0.841 ± 0.082; *p* = 0.048). This value was significantly higher than that observed in the UT group POST (*UT* 0.701 ± 0.167, *TR* 0.841 ± 0.082; *p* = 0.011). Additionally, APAs iEMG of the TrA/IO in the UT group exhibited a significant decline after fatigue (*PRE* 0.712 ± 0.173, *POST* 0.597 ± 0.221; *p* = 0.039). 

### 3.4. Co-Contraction Indices of Muscle Pairs

**Figure 4:**
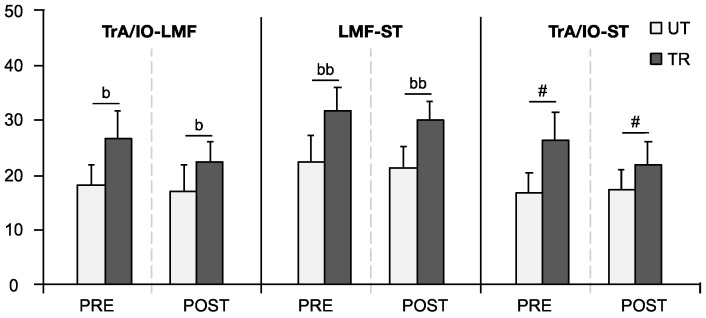
Co-activation indices of muscle pairs for APAs during self-initiated perturbation. Data are presented as mean ± SE. ‘#’ indicates a significant main group effect (*p* < 0.05). ‘b’ signifies a significant difference between trained (TR) and untrained (UT) groups (*p* < 0.05), ‘bb’ (*p* < 0.01).

As shown in Figure 4, the Wilcoxon test indicated that the APA co-contraction indices of TrA/IO-LMF and LMF-ST in the TR group were significantly higher than those in the UT group, both in PRE (TrA/IO-LMF: *UT* 18 ± 8.1, *TR* 26.5 ± 11.1, *p* = 0.011; LMF-ST: *UT* 22.5 ± 9.9, *TR* 31.6 ± 9.6, *p* = 0.002) and POST conditions (TrA/IO-LMF: *UT* 16.9 ± 10.2, *TR* 22.4 ± 7.6, *p* = 0.029; LMF-ST: *UT* 21.1 ± 8.8, *TR* 29.9 ± 7.6, *p* = 0.005). The APAs co-contraction index of TrA/IO-ST demonstrated a significant main group effect (F_(1,34)_ = 6.779, *p* = 0.014, $\eta_{p}^{2}$ = 0.166), with the TR group recording significantly higher values than the UT group.

**Figure 5:**
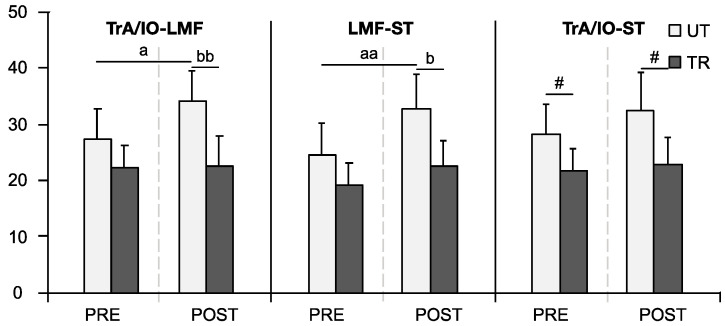
Co-activation indices of muscle pairs for CPAs during self-initiated perturbation. Data are presented as mean ± SE. ‘a’ denotes a significant difference between pre-(PRE) and post-fatigue (POST) conditions (*p* < 0.05), ‘aa’ (*p* < 0.01). ‘b’ signifies a significant difference between trained (TR) and untrained (UT) groups (*p* < 0.05), ‘bb’ (*p* < 0.01). ‘#’ indicates a significant main group effect (*p* < 0.05).

For the CPA co-contraction indices, the UT group showed significant increases after fatigue for TrA/IO-LMF and LMF-ST (TrA/IO-LMF: *PRE* 27.5 ± 11.4, *POST* 34.4 ± 11.7, *p* = 0.035; LMF-ST: *PRE* 24.6 ± 12.2, *POST* 32.9 ± 13.2, *p* = 0.005). POST, these values were significantly higher in the UT group compared to the TR group (TrA/IO-LMF: *UT* 34.4 ± 11.7, *TR* 22.7 ± 11.5, *p* = 0.004; LMF-ST: *UT* 32.9 ± 13.2, *TR* 22.7 ± 9.8, *p* = 0.020). Additionally, a significant main group effect was noted in the CPAs co-contraction index of TrA/IO-ST (F_(1,34)_ = 5.802, *p* = 0.022, $\eta_{p}^{2}$ = 0.146), with the UT group exhibiting a higher index than the TR group, as illustrated in Figure 5. 

### 3.5. Center of Pressure

**Figure 6:**
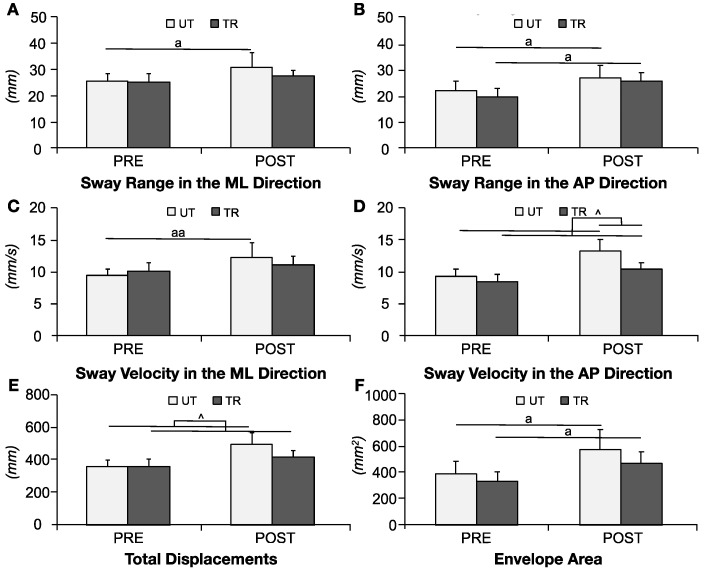
Variables of center of pressure during postural stability test. (**A**) ML-sway range; (**B**) AP-sway range; (**C**) ML-sway velocity; (**D**) AP-sway velocity; (**E**) Total displacements; (**F**) Envelope area. Data are presented as mean ± SE. ‘a’ indicates a significant difference between pre-(PRE) and post-fatigue (POST) conditions (*p* < 0.05), ‘aa’ (*p* < 0.01). ‘^’ denotes a significant group-by-fatigue interaction effect (*p* < 0.05).

Outcomes for the COP are depicted in Figure 6. The Wilcoxon test indicated significant increases in both the ML-sway range (*PRE* 25.4 ± 6.3, *POST* 31 ± 11.8, *p* = 0.028) and velocity (*PRE* 9.3 ± 2.1, *POST* 12.1 ± 4.7, *p* = 0.004) for the UT group after fatigue. Both the UT (*PRE* 21.8 ± 8.6, *POST* 26.9 ± 10.2, *p* = 0.035) and TR (*PRE* 19.6 ± 7.1, *POST* 25.6 ± 7.1, *p* = 0.016) groups demonstrated an expanded AP-sway range after fatigue. For AP-sway velocity, significant main effects for group (F_(1,34)_ = 4.605, *p* = 0.039, $\eta_{p}^{2}$ = 0.119) and condition (F_(1,34)_ = 42.457, *p* < 0.001,$\eta_{p}^{2}$ = 0.555) were observed, along with a significant group-by-condition interaction effect (F_(1,34)_ = 5.217, *p* = 0.029, $\eta_{p}^{2}$ = 0.133). Simple effect analysis revealed that both groups experienced an increase in AP-sway velocity after fatigue (UT: *PRE* 9.1 ± 2.5, *POST* 12.9 ± 3.9, *p* < 0.001; TR: *PRE* 8.3 ± 2.2, *POST* 10.1 ± 2.2, *p* = 0.005), with the UT group exhibiting a significantly higher AP-sway velocity than the TR group POST (*p* = 0.013). 

Total displacement demonstrated a significant main effect for condition (F_(1,34)_ = 26.631, *p* < 0.001, $\eta_{p}^{2}$ = 0.439) and a significant group-by-condition interaction effect (F_(1,34)_ = 4.155, *p* = 0.049, $\eta_{p}^{2}$ = 0.109). Simple effect analyses indicated that both the TR (*PRE* 359 ± 95.9, *POST* 416 ± 96.3, *p* = 0.034) and UT (*PRE* 363.3 ± 83.7, *POST* 494.8 ± 153.7, *p* < 0.001) groups showed significantly increased total displacements after fatigue. The Wilcoxon test also revealed significant increases in the envelope areas for both the TR (*PRE* 339.4 ± 151.3, *POST* 475.1 ± 201.2, *p* = 0.039) and UT (*PRE* 391.5 ± 208, *POST* 577.7 ± 334.9, *p* = 0.018) groups after fatigue.

## 4. Discussion

This study examined the effects of general fatiguing exercises on APAs, CPAs, and postural stability between individuals with a comprehensively trained status and those without any sports training experience. The results indicate: (1) Regardless of fatigue conditions, the APAs onset of the TrA/IO, LMF, and ST muscles was significantly earlier in the TR group than in the UT group; (2) Moreover, within the APAs phase, the co-contraction indices of the TrA/IO-LMF, TrA/IO-ST, and LMF-ST muscle pairs were significantly higher in the TR group than in the UT group; (3) Within the CPAs phase, the UT group demonstrated a significant increase in co-contraction indices of the TrA/IO-LMF and LMF-ST muscle pairs after fatigue, significantly greater than that in the TR group; (4) Fatiguing exercise impacted the postural stability of both groups. However, the UT group exhibited significantly greater sway across several COP indicators than the TR group after fatigue.

### 4.1. Enhanced APAs Patterns in the TR Compared to the UT Group

APAs, as reflected by the surface electromyography of postural muscles, are considered manifestations of central feedforward control. Research has noted APA onset delays under conditions such as lower back pain, aging, and exhaustive fatigue, accompanied by reduced postural stability [26]. Conversely, certain studies report that, following moderate fatigue, the onset of APAs in some postural muscles significantly advances [27]. The Similar Impulse Hypothesis [10] and the Critical Force Hypothesis [11] propose that the early onset of APAs in postural muscles represents a localized protective strategy by the central nervous system. This strategy involves extending the preparatory phase for postural muscles to maintain force levels comparable to pre-fatigue conditions, thereby mitigating the risk of posture instability following fatigue interventions. Therefore, an earlier onset of APAs may provide a safer and more efficient response to predictable disturbances. 

Research comparing APAs and CPAs between professional athletes and non-athletes is limited. Existing studies have observed that professional archers exhibit anticipatory postural sway (COP-APAs) prior to arrow release, a response not seen in novices. Moreover, while the APA activations of the RF are greater in professionals, the APA activations of the TA are more pronounced in novices, suggesting proximal versus distal compensation strategies, respectively [28]. Another study indicates that expertise in fencing delays TA activation and COP displacement during a lunge compared to controls. Fencers demonstrate typical APA activation patterns in the TA across various performance conditions, conflicting with many previous studies. Researchers suggest that the high demands of fencing, requiring rapid and flexible responses, may lead to adaptations where APAs do not necessarily initiate earlier [1]. Thus, specialized sports training may not uniformly enhance the training effects on APAs and CPAs. The impact of different sports on APAs and CPAs remains to be further explored.

Many studies have reported that both healthy individuals and patients exhibit significantly earlier onsets of muscle APAs following brief periods of specific training, such as a single session of functional exercise (lasting 20–25 min) [29], three-day repeated training [30], four-week functional training [31], and eight weeks of boxing training [19]. These findings indicate that exercise interventions involving repeated exposure to movements akin to those in postural disturbance tests can significantly advance the onset of APAs [29].

However, as previous research has suggested, the benefits of exercise training on postural control are movement-specific and barely transfer to untrained or inexperienced postural and motor tasks [16]. Therefore, this study included athletes with a comprehensive foundational training experience—that is, at least two years of training focused on speed, strength, power, and endurance—but who had not specialized in a single sport. We observed that the trunk and lower limb muscles of the TR group consistently exhibited earlier APA onsets compared to the UT group, regardless of fatigue state. This suggests that continuous, comprehensive training focused on speed, strength, power, and endurance significantly alters the human body’s response patterns to predictable interventions, characterized by earlier APA activation times and greater co-activation amplitudes, with this response pattern being almost unaffected by fatigue in our experimental setup. We suggest that comprehensive physical training may enhance sensory sensitivity, improve the efficiency of central information transmission, integration, and processing, update internal models of movement control, and enhance muscle contraction functionality, thus altering APA response patterns. Future research could increase fatigue intensity to observe APA response patterns in more challenging scenarios among trained individuals.

### 4.2. Interaction of Muscle Adjustments with Postural Stability in Different Groups

The timing and intensity of APAs in postural muscles are complementary to maintaining human postural stability. In our findings, the TR group consistently demonstrated superior APA strategies than the UT group, characterized by an earlier onset of the ST muscle and greater iEMG responses following fatiguing exercise. The hamstring plays a critical role in balance by stabilizing the hip joint during standing, thereby enhancing balance in the sagittal plane [32]. Hence, the effective activation of the hamstring in the TR group likely contributed to reduced AP-sway during quiet standing, aligning with our findings on postural stability. The TrA and IO muscles, categorized as deep muscles, are local core muscles that directly impact segmental spinal stability. Ferraro R, Garman S, Taylor R, et al. [33] discovered that training to improve neuromuscular control of the TrA significantly influenced the control of the COP in the ML direction. Our study also found that the UT group exhibited reduced TrA/IO APAs iEMG during self-initiated perturbation tests post-fatigue and increased ML-sway in postural stability tests, a phenomenon not observed in the TR group.

Coordinated activation of the abdominal flexors and extensors is one of the fundamental neural mechanisms for maintaining spinal stability [34]. Therefore, changes in their activation patterns can potentially affect postural performance. Previous studies suggest that muscle co-contraction is a relatively less efficient but safer strategy, particularly common among individuals with neurological conditions and the elderly [29], with research finding greater muscle co-contraction at the ankle in older adults compared to younger individuals [35].

Intriguingly, our research found that irrespective of PRE or POST conditions, the TR group’s APA co-contraction indices for the TrA/IO-LMF, LMF-ST, and TrA/IO-ST muscle pairs were higher than those of the UT group. Only in the CPA phase did the UT group exhibit co-contraction indices that were higher than those of the UT group after fatigue. Furthermore, the differences in APAs and CPAs co-contraction indices between the TR and UT groups were more pronounced than those in individual muscle iEMG.

Consequently, we propose that the TR group adopts a more efficient, patterned activation strategy when confronted with self-initiated perturbations, involving earlier activation times and greater co-contraction intensity of trunk and lower leg stabilizing muscles. This likely results from sensory-motor adaptability changes caused by continuous stimuli from intense, comprehensive training.

### 4.3. Limitations

A limitation of this study is the inability to simultaneously measure changes in the COP during self-initiated postural perturbation tests. Consequently, the correlation and causality between muscle activity during disturbances and static balance performance could not be further confirmed in this study.

Another limitation of this study is the use of a 20-min rowing exercise as a fatigue intervention, which complicates the quantification of fatigue levels. Particularly, significant differences in resting heart rates between TR and UT, along with varying fatigue tolerance, lead to inconsistent changes between HR and RPE between groups. Additionally, since rowing is a general exercise, assessing fatigue levels solely through electromyographic signals from a single muscle presents challenges. Future studies should seek more quantifiable and standardized methods for measuring fatigue interventions.

## 5. Conclusions

This study assesses the impact of general fatiguing exercises on APAs, CPAs, and postural stability between individuals with comprehensively trained status and those without sports training experience. The findings show that the TR group consistently demonstrates an efficient APA pattern, marked by early activation and enhanced co-contraction of APAs in the trunk and lower leg postural muscles, regardless of fatigue. Additionally, the study reveals that the UT group shows increased CPA co-contraction in trunk stabilizer muscles after fatigue, indicating a lack of sufficient anticipation and a relatively passive response to self-initiated postural disturbances, associated with a heightened risk of falls and injuries during exercise when fatigued. Overall, this study underscores the importance of integrating comprehensive training programs to enhance postural and neuromuscular responses, especially for populations at an elevated risk of postural instability.

## Figures and Tables

**Table 1:** Demographic and training information for trained (TR) and untrained (UT) groups.

Group	TR	UT
Age (years)	22.2 ± 0.7	22.6 ± 1.2
Height (cm)	178.0 ± 6.0	176.0 ± 4.0
Weight (kg)	69.2 ± 7.4	67.3 ± 8.1
Body mass index (kg/m^2^)	21.8 ± 1.6	22.0 ± 2.7
Training years (years)	3.8 ± 3.3	0

**Table 2:** Ratings of perceived exertion (RPE) and heart rate (HR) for trained (TR) and untrained (UT) groups pre- (PRE) and post-fatigue (POST).

Group	TR			UT		
Condition	PRE	POST	Percentage Change (%)	PRE	POST	Percentage Change (%)
RPE	10.1 ± 1.7	14.5 ± 1.8	43.56%	7.7 ± 1.7	16.6 ± 2.7	115.58%
HR (bpm)	65.2 ± 6.3	135.4 ± 20.4	107.67%	80.4 ± 11.0	148.3 ± 16.2	84.45%

## Data Availability

The data presented in this study are available on request from the corresponding author. The data are not publicly available due to ethical reasons.
